# Operational Strategies for Clinical Trials in Africa

**DOI:** 10.1200/JGO.19.00204

**Published:** 2020-07-02

**Authors:** Katy M. Graef, Ifeoma Okoye, Naomi O. Ohene Oti, Jennifer Dent, Folakemi T. Odedina

**Affiliations:** ^1^ BIO Ventures for Global Health, Seattle, WA; ^2^ Department of Radiology, College of Medicine, University of Nigeria, Nsukka, Nigeria; ^3^ University of Nigeria Centre for Clinical Trials, University of Nigeria Teaching Hospital, Enugu, Ituku Ozalla, Nigeria; ^4^ National Centre for Radiotherapy and Nuclear Medicine, Korle Bu Teaching Hospital, Accra, Ghana; ^5^ University of Florida, Orlando, FL; ^6^ Prostate Cancer Transatlantic Consortium, Orlando, FL

## Abstract

**PURPOSE:**

In a dramatic reversal of longstanding trends, cancer now kills more Africans than malaria. Despite Africa’s growing cancer burden, individuals of African descent, notably those residing in Africa, remain drastically under-represented in cancer clinical trials. Two recent summits—the 1st All Africa Clinical Trial Summit and the Operational Strategy for Clinical Trials in Nigeria Summit—convened experts from governments, the private sector, universities, and professional societies to define the barriers to Africa’s participation in multicenter clinical studies and the strategies to eliminate those impedances.

**METHODS:**

The discussions held during the two clinical trial summits were condensed into a set of 10 recommendations covering five broad categories (funding, regulation, capacity building, Africa-centric approach, and patient engagement). In this article, four programs are presented as examples of how the summits’ recommendations can be put into practice to improve Africa’s ability to attract clinical trials, in particular, cancer clinical trials.

**RESULTS:**

These example programs all leveraged a multilateral, Africa-driven approach to building Africa’s clinical trial capacity, increasing visibility of Africa’s current clinical trial capabilities and priorities, improving regulatory infrastructure and enforcement on the continent, and optimizing patient and clinician engagement strategies.

**CONCLUSION:**

The four programs are anticipated to catalyze the involvement of more African health care sites in cancer clinical trials, enroll a greater number of African patients with cancer in those trials, and, ultimately, reverse Africa’s growing cancer incidence and mortality rates. Each program acts as a blueprint for organizations—whether government, academic, or industry—seeking to address the summits’ recommendations and increase Africa’s contributions to and active participation in clinical research.

## INTRODUCTION

Clinical trials are integral to the development of safe and effective medical treatments and technologies and are necessary to improving clinical care outcomes and identifying solutions to disease management challenges. These studies are also an essential cancer treatment modality, with the National Comprehensive Cancer Network noting that the best option to manage patients with cancer is through clinical trials.^1^ Despite this recommendation, a disproportionately higher number of white patients enroll in cancer clinical trials compared with other ethnicities. In 2018, the US Food and Drug Administration estimated that only 4% of patients enrolled in clinical trials that led to the approval of new cancer drugs were of African descent.^2^ Similarly, of the 68,673 cancer studies listed in Clinical Trials.gov, < 2% (917) were conducted on the African continent.^3^ Of these 917 studies, the majority were performed in South Africa and Egypt.^4^

**Context**

**Key Objective**
Are cancer clinical trials in Africa possible, and, if so, what can be done to encourage the engagement of African clinical sites in cancer studies?
**Knowledge Generated**
Cancer clinical trials are, to a limited extent, underway in Africa, but the involvement of a greater number of sites and countries is needed to adequately assess new cancer innovations across all African ethnicities. Programs are underway to map and build clinical trial capabilities in Africa, with the goal of increasing the number of clinical trials performed on the continent.
**Relevance**
With Africa’s cancer mortality rate predicted to double by 2040, more clinical trials on important cancer innovations can—and must—be conducted in Africa.

Despite these statistics, individuals of African descent, in particular those living on the African continent, are disproportionately affected by cancer. The age-standardized incidence rates of Kaposi’s sarcoma are ten times higher, cervical cancer rates are four times higher, and liver cancer rates are 24% higher in Africa than in the United States.^5^ These three cancers are linked to infectious agents and are more prevalent in HIV-positive individuals.^6,7^ Beyond HIV/AIDS-associated malignancies, women of African descent are diagnosed with higher rates of triple-negative breast cancer than other ethnicities.^8^ Men of African descent are disproportionately affected by prostate cancer and have poorer outcomes.^9^ The risk of dying as a result of cancer before the age of 75 years in Africa is nearly double that of the risk to individuals living in a high development index country.^10,11^

These statistics are the result of many complex and interrelated factors, including inadequate health care systems in Africa and African ethnicities’ unique, intrinsic tumor genetics and biologies.^12^ Although cancer risk, incidence, and survival are known to differ across ethnicities, more troubling are recent predictions that demonstrate an inverse correlation between the drug resistance and susceptibility of lung cancers from patients of European and African descent.^13^ Clinical trials with subjects of diverse racial and ethnic backgrounds are thus imperative to ensuring that new cancer treatments are equally safe and efficacious across all patients with cancer.

In recognition of Africa’s limited involvement in clinical trials, two summits—the 1st All Africa Clinical Trial Summit and the Operational Strategy for Clinical Trials in Nigeria Summit—were held to define Africa’s clinical trial capabilities and gaps and conceptualize strategies to encourage the performance of clinical trials on the continent. Both meetings were attended by key opinion leaders and experts from government health, science, technology, and regulatory agencies; the private sector; universities; and professional societies from seven countries (Table 1). These experts presented their experiences and insights conducting clinical trials in Africa and globally and engaged in panel discussions and workshops to dissect the challenges of conducting clinical trials on the African continent and to explore strategies to eliminate these challenges.

**TABLE 1:**
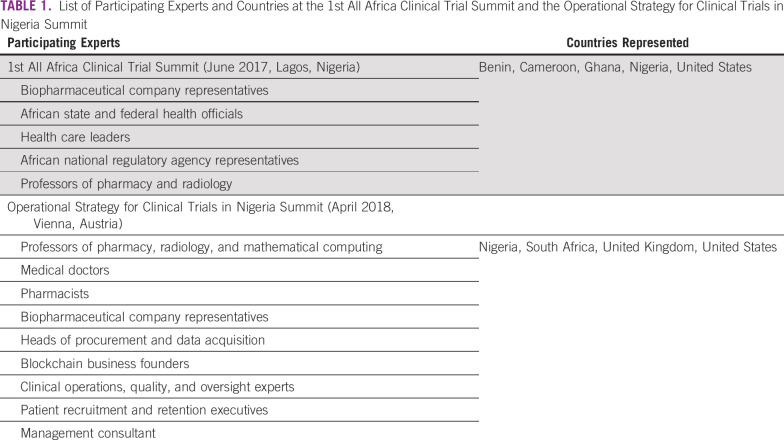
List of Participating Experts and Countries at the 1st All Africa Clinical Trial Summit and the Operational Strategy for Clinical Trials in Nigeria Summit

After each summit, the organizers summarized the participants’ observations, experiences, and recommendations and published these findings in proceedings reports.^14,15^ We have reviewed these proceedings reports, organized the expert recommendations on the basis of their underlying themes, and distilled the themes and recommendations into five broad categories: funding, regulation, capacity building, Africa-centric approach, and patient engagement (Table 2). This article presents four programs as examples of how the summits’ recommendations can be put into action to achieve better representation of the African continent in global clinical trials. Although not a comprehensive list of all clinical trial programs underway in Africa, these four programs offer examples of how organizations seeking to improve Africa’s participation in clinical research can get involved and make an impact.

**TABLE 2:**
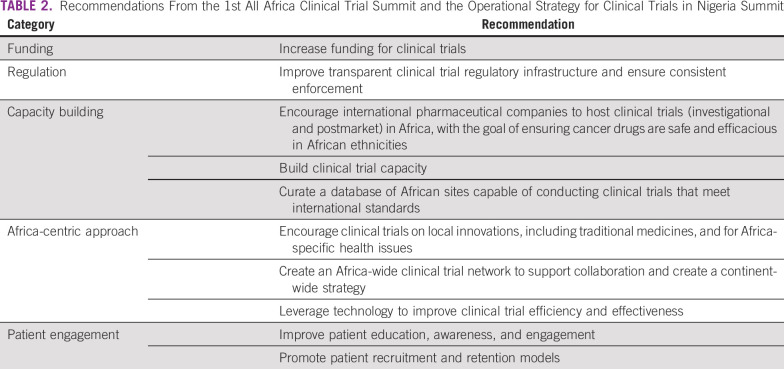
Recommendations From the 1st All Africa Clinical Trial Summit and the Operational Strategy for Clinical Trials in Nigeria Summit

### African Consortium for Cancer Clinical Trials

In partnership with African ministries of health and cancer hospitals, private industry, and global oncology experts, BIO Ventures for Global Health (BVGH) launched the African Consortium for Cancer Clinical Trials (AC^3^T) to evaluate African cancer hospitals’ clinical trial “readiness” and promote the hospitals’ capabilities to private industry and international academic investigators.

With the input and guidance of its partners, BVGH developed a self-assessment tool (AC^3^T Checklist) to evaluate African hospitals’ management capabilities for clinical trials and patients with cancer (Table 3) and to compare these capacities against international standards. The data obtained from a hospital’s checklist are also used to populate a hospital-specific profile, which is published on BVGH’s online AC^3^T platform. The online platform, which launched in January 2020, is accessible to private industry and academic investigators.^16^ The goal of the online platform is to help guide investigators’ search for African clinical trial sites and to narrow down the list of potential sites for subsequent due diligence and on-site assessments.

**TABLE 3:**
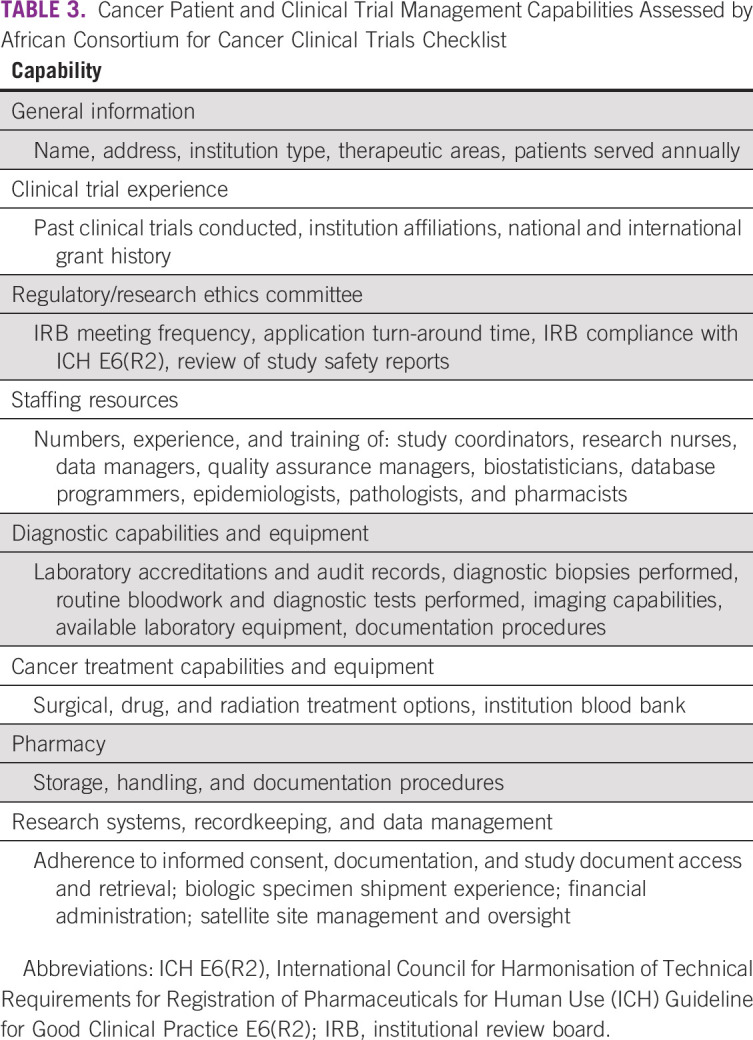
Cancer Patient and Clinical Trial Management Capabilities Assessed by African Consortium for Cancer Clinical Trials Checklist

Before launching the AC^3^T Checklist continent-wide, BVGH piloted its use across five African countries: Cameroon, Côte d’Ivoire, Kenya, Nigeria, and Rwanda. These five countries were selected based on their federal governments’ prioritization of cancer and participation in another BVGH program, the African Access Initiative, which focuses on improving outcomes for patients with cancer in Africa.^17^ BVGH distributed the AC^3^T Checklist to 24 cancer hospitals, which had been selected by their respective ministries of health to participate in the African Access Initiative. These hospitals are geographically evenly distributed, where possible, across the five countries. Reflecting the typical locations for cancer treatment in these countries, the hospitals assessed are located in urban centers.

To date, 15 of the 24 hospitals have returned their checklists (Appendix Table A1). On reviewing the returned AC^3^T Checklists, BVGH identified several areas for the assessment tool’s improvement—namely its format (Word document), length, questions, and language. The checklist has since been reformatted (PDF), and questions whose responses would be of limited use to external investigators were removed. The checklist is also being translated into French and Portuguese to expand its accessibility to Africa’s francophone and Lusophone countries. Additional refinement to the tool will be made as BVGH receives additional checklists from sites across Africa.

With the pilot complete, BVGH has begun distributing the updated checklist to a wider array of hospitals and clinical research sites, including institutions within the European and Developing Countries Clinical Trials Partnership (EDCTP) networks of excellence. BVGH is also broadening its outreach to institutions without cancer capacities in an effort to increase the number of sites assessed. Although not currently suitable for clinical trials assessing novel cancer treatments, these sites could host studies to assess new cancer diagnostics and vaccines as well as behavioral and epidemiologic studies.

In addition to promoting hospitals’ clinical trial proficiencies, the AC^3^T Checklist also highlights hospitals’ clinical infrastructure, processes, and human resources deficits. Using the results of the AC^3^T Checklists, BVGH has begun organizing training projects customized to address responding hospitals’ core competency gaps. Each training project leverages BVGH’s alliance and program management experience and the oncology expertise of its partners. For example, BVGH’s assessments revealed that a hospital in Côte d’Ivoire required additional training, laboratory workflow management, and capital investment to improve its diagnosis of patients with cancer. Working with the American Society for Clinical Pathology, BVGH organized a workshop, fellowship, and equipment placement at the Côte d’Ivoire hospital. Each of these activities was specifically undertaken to help the hospital eliminate a competency gap and achieve the standards of diagnosis necessary for the conduct of cancer clinical studies.

### Prostate Cancer Transatlantic Consortium

The Prostate Cancer Transatlantic Consortium (CaPTC) is a National Institutes of Health/National Cancer Institute Epidemiology and Genomics Research Program–supported consortium founded in 2005 to address the complexity of cancer health disparities and the need for a unique approach to better understand and address complex chronic diseases.^18^ As a global consortium, CaPTC has > 150 members from 15 countries, including a significant presence in Africa. Members include prostate cancer clinicians, scientists, and advocates with complementary expertise and resources who are working collaboratively to eliminate the disproportionate burden of prostate cancer globally. The primary goal of CaPTC is to explore the genetic and environmental etiology of prostate cancer and to develop ethnically sensitive, targeted approaches that will eliminate prostate cancer disparities globally in men of African descent.

To achieve the primary goal of CaPTC, the consortium has set a 2020 goal of enhancing the engagement of individuals of African descent in cancer clinical trials. The challenges of conducting cancer clinical trials in Africa are multifaceted and formidable, with the most significant challenge being the lack of cancer clinical trial expertise on the continent. CaPTC has developed and implemented a series of clinical trial workforce development programs to build a cohort of African cancer leaders who can make a significant impact on clinical trials research, education, and training in Africa. The training programs are varied to meet the needs of Africa’s oncology workforce. In late 2018, CaPTC implemented a webinar series targeting African investigators and clinicians. The series covered a range of topics, including the practical steps to implementing clinical trials, the role of clinical research organizations, patient advocacy, tips for successful clinical trials in Africa, and operational considerations and technology innovations for clinical trials.

In partnership with the University of Cape Town, CaPTC organized the African Oncology Clinical Trials (AfrOCT) Leadership Training and Development Program. The AfrOCT program aims to implement a didactic training program on the design and funding of clinical trials and to provide experiential training on the execution and management of clinical trials. The learning objectives of the program are to: (1) instill an understanding of clinical trial management processes, inclusive of critical documentation required for efficiency; (2) provide on-site experiential training and link participants with an assigned mentor; (3) provide information on potential business models for clinical trial units; (4) discuss methods for overcoming perceived barriers and challenges expressed by trainees; and (5) develop short- and long-term performance indicators with delegates for program evaluation and future training needs.

**FIG 1:**
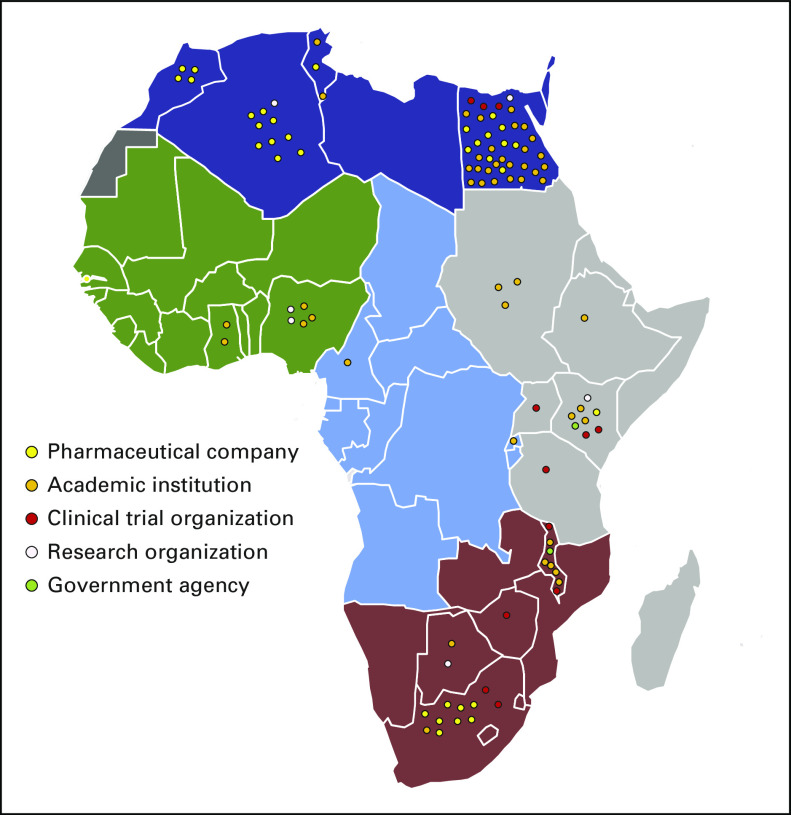
Map of ongoing cancer clinical trials in Africa. Circles represent individual clinical trials, with the color of the circles denoting the study’s primary sponsor type.

Beyond organizing training opportunities for African clinical research professionals, CaPTC has also focused on improving clinicians’ and patients’ access to clinical trials in Africa by developing a virtual cancer clinical trial map. With funding from the Carnegie Corporation, CaPTC investigators created a database of African cancer clinical trials infrastructure, resources, and research sites. As a part of this project, a study was performed to identify ongoing cancer clinical trials in Africa. The quantitative, web-based, retrospective review of clinical trial registries documented a total of 109 open cancer clinical trials in Africa (Figure 1), with most of the trials located in Egypt, South Africa, Algeria, and Kenya. The top cancer types under assessment were breast, cervical, and lung. The top sponsors of oncology clinical trials in Africa were academic institutions, especially institutions based in the United States. Using these data, CaPTC has launched a website that captures information on open cancer clinical trials and allows investigators to register new studies.^19^ CaPTC is currently developing and testing the usability of its Oncology Clinical Trials AppConnect (OnCTAC), which will help African patients and health care professionals search for cancer clinical trials and connect with clinical trial staff via a smartphone application.

Achieving international good clinical practice (GCP) standards is a necessary checkpoint for a site seeking to conduct clinical trials, and yet in Africa there is incomplete compliance with GCP standards, such as those for subject recruitment and enrollment.^20^ CaPTC developed the African Community Cancer Research Engagement and Support (CCaRES) Network to facilitate linkages between researchers and clinical trial participants and improve adherence to GCP standards. The African CCaRES Network is composed of a participant data warehouse and a scientist information database linked by CCaRES informatics and provides a community-centric system that will educate and direct participants to appropriate, institutional review board (IRB)–approved biomedical research. Five services will be provided through the African CCaRES Network: clinical trial education, enrollment, navigation, matching, and monitoring/follow-up services. The African CCaRES Network will use best practices for patient recruitment, enrollment, and retention in Africa and collect data on and continuously address individual-level barriers that influence clinical trial participation in Africa.

### Association for Good Clinical Practice in Nigeria and African Clinical Trial Consortium

Established in 2006 and hosted by the University of Nigeria, Nsukka, Enugu campus, the Association for Good Clinical Practice in Nigeria (AGCPN) seeks to encourage the performance of clinical trials in Nigeria by supporting the training of clinical research professionals, advocating for the improvement of Nigeria’s clinical trial infrastructure, increasing global visibility of Nigeria’s clinical trial capabilities, and creating a coordinated and collaborative network of clinical research professionals.

AGCPN conducts annual in-person and in-house trainings in GCP, health research ethics, and biostatistics. These trainings are complemented by online educational opportunities offered through a partnership with the University of Miami’s Collaborative Institutional Training Initiative. With funding from EDCTP, AGCPN has also provided certified health research ethics training for 36 IRB members from 18 institutions across Nigeria. AGCPN’s trainings focus on introducing local content germane to African culture, especially as the trainings relate to study subject engagement.

As Nigeria’s premier organization responsible for shaping and driving the development of the country’s clinical trial system, AGCPN has built relationships with relevant government agencies and parastatals and provides those entities with guidance and assistance. In particular, AGCPN has supported Nigeria’s National Agency for Food and Drug Administration and Control (NAFDAC) by assisting the agency with the revision of its clinical trial regulations (2010), performing a gap analysis of NAFDAC’s clinical trial unit (2012), and supporting NAFDAC’s launch of the Nigerian Clinical Trial Technical Working Group (2015). AGCPN has also been leading advocacy efforts in Nigeria for the introduction of improved research facilities, multidisciplinary professional infrastructures, standards for GCP designed to protect clinical trial participants, and provision of rigorous and robust clinical trial regulations.

Emulating China and India’s strategy of increasing their global visibility through clinical trial summits, AGCPN also launched its first Clinical Trial Summit in 2012. The event convened clinical research professionals from across Nigeria for education and networking opportunities, as well as to highlight the clinical trials ongoing and concluded in Africa’s most populous country.

Noting that the African clinical trial sector needs to apply a coordinated, collaborative, continent-wide strategy that mirrors the African Union vision for a developed and economically integrated Africa, AGCPN launched the AGCPN–Clinical Trial Africa (CTA) Vision 2020 Initiative. With signatories from Cameroon, Benin, Ghana, and Nigeria, this initiative was established in 2016 to provide a platform for sustained advocacy, networking, and engagement of relevant stakeholders to nurture African development of African solutions to Africa’s health challenges for both communicable and noncommunicable diseases. The Initiative catalyzes dialogue between sponsor organizations, companies, African governments, and other relevant influencers and led to the formation of the African Clinical Trial Consortium (ACTC) in 2017.

Similar to the AGCPN-CTA Vision 2020 Initiative, the mission of ACTC is to promote, nurture, and sustain a continent-wide advancement of health research through the efficient use of local knowledge garnered from the public and private sectors. ACTC focuses on addressing the low number of clinical trials occurring in Africa and developing innovative models for the certification and strategic oversight of clinical trial sites on the continent. ACTC has prioritized the creation of dedicated clinical trial units in Africa and the growth of Africa’s natural medicinal product development. The latter effort was fostered by engaging with Nigeria’s Natural Medicine Development Agency within the Federal Ministry of Science and Technology, natural medicine developers and practitioners, and research clinicians to explore investigator-initiated studies on Nigerian natural products.^21^ Beyond the rigorous assessment of natural medicines, other anticipated benefits of ACTC include the expansion of Africa’s cost-effective clinical capabilities that meet global standards, acceleration of clinical trial timelines in Africa, incorporation of a translational research mentality into Africa’s research institutions, and additional definition and institutionalization of the continent’s regulatory systems.

AGCPN’s efforts in the African clinical trial ecosystem have spanned over two decades and have focused on building the infrastructure for global engagement of Nigeria in single-center and multicenter clinical trials. Through its resultant initiatives, AGCPN-CTA Vision 2020 Initiative and ACTC, AGCPN’s impact has extended beyond Nigeria’s borders. In time, it is expected that additional countries beyond the initial four signatories will join AGCPN’s initiatives, thereby expanding their trainings continent-wide.

### Building Clinical Trial Regulation and Oversight Infrastructure in Ghana

**FIG 2:**
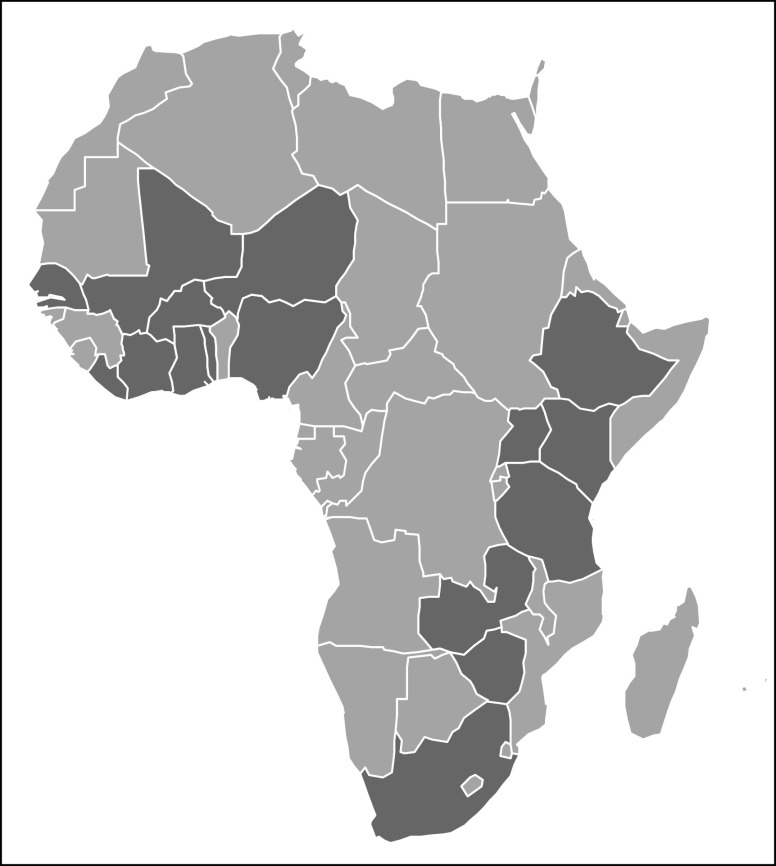
Map of African countries with medicines review agencies that have clinical trial oversight authority. Review agencies that have clinical trial oversight authority are noted in dark gray. Although not shown on the map, the Cabo Verde’s medicines review agency has clinical trial oversight authority. Records demonstrating the clinical trial oversight authority of the medicines review agencies of the countries shown in light gray are unavailable.

Proper regulation and oversight are essential to ensuring clinical trials are conducted with the highest standards of care and achieve clinically relevant and actionable data. All African countries, with the exception of the Sahrawi Arab Democratic Republic, have functioning medicine review agencies. However, their authorities to regulate clinical trials within their countries are varied. Only 17 of 55 African countries have regulatory agencies with control over clinical trials (Figure 2).^22^ If African cancer hospitals are to attract international cancer clinical trials, regulatory frameworks must be in place at the institutional and the national level. African countries without such oversight should take advantage of multinational entities, such as the Pan African Clinical Trial Registry and EDCTP, as well as leverage the experiences and learnings of their nearest neighbors to guide their installation of clinical trial oversight authorities. Ghana’s introduction of regulations and oversight authorities into its national health and research systems is a strategy that other African countries can emulate as they begin to adopt their own clinical research regulatory systems.

Clinical studies have been reported in Ghana since the 1960s, with the earliest interventional study reported in 1968.^23^ The majority of clinical trials performed in Ghana to date have focused on communicable disease interventions, such as the 1968 study, which assessed chloroquine resistance in malaria parasites circulating in Axim, Ghana.^23,24^ Oncology-related clinical trials were first recorded in the 1970s and focused on childhood Burkitt lymphoma, including a study on high-dose cyclophosphamide to treat resistant and relapsing Burkitt lymphoma.^25^

These clinical studies were implemented despite the absence of rigorous nationwide or institutional clinical trial regulations and ethics guidelines. In 1992, the Ghanaian Ministry of Health established the Food and Drug Board (FDB) through the Food and Drugs Act (provisional National Defence Council law 3058) to regulate clinical trials in Ghana.^26^ Subsequent to this, the Noguchi Memorial Institute for Medical Research (a semiautonomous branch of the University of Ghana) and Navrongo Health Research Center established their IRBs.^27,28^ Additional research institutions and universities eventually began to establish their own IRBs. In 2012, the Ghanaian Legislature further reviewed the Food and Drugs Act and incorporated it into a new law (Public Health Act 851), which broadened the FDB’s clinical trial regulatory activities and transformed the FDB into the Food and Drug Authority (FDA).^29,30^ The FDA currently regulates all clinical trial activities in the country and must issue a clinical trial authorization certificate before a clinical study can commence. To receive the certificate from the FDA, the principal investigator, who must be a Ghanaian, must register the study with Pan African Clinical Trial Registry and receive IRB approval from the facility or site at which the study will be conducted.

In concert with the country’s clinical trial policy changes, Ghana began implementing training programs on GCP. Before this, there were minimal opportunities to receive GCP training in the country; most research institutions opted to send their clinical trial staff abroad for the training. Now, as a component of its mandate, the FDA conducts GCP trainings, including “train the trainer” programs. The FDA also now requires that all clinical research teams have GCP certificates before a clinical trial authorization certificate will be issued.

## DISCUSSION

The four programs described above collectively address many of the challenges identified by the participants of the 1st All Africa Clinical Trial Summit and the Operational Strategy for Clinical Trials in Nigeria Summit (Table 4). In particular, AC^3^T is mapping clinical trial capabilities across Africa and promoting those capabilities to external investigators. AC^3^T, AGCPN, and CaPTC are building clinical trial capacity in Africa, while also creating continent-wide clinical trial networks. Through ACTC, AGCPN is building an environment in Nigeria that encourages rigorous clinical assessments on traditional medicines. CaPTC’s OnCTAC is helping connect African patients with cancer and health care providers with open clinical trials. Last, Ghana’s process of establishing a clinical trial regulatory system provides a roadmap for other African countries seeking to improve the regulation of clinical trials within their borders.

**TABLE 4:**
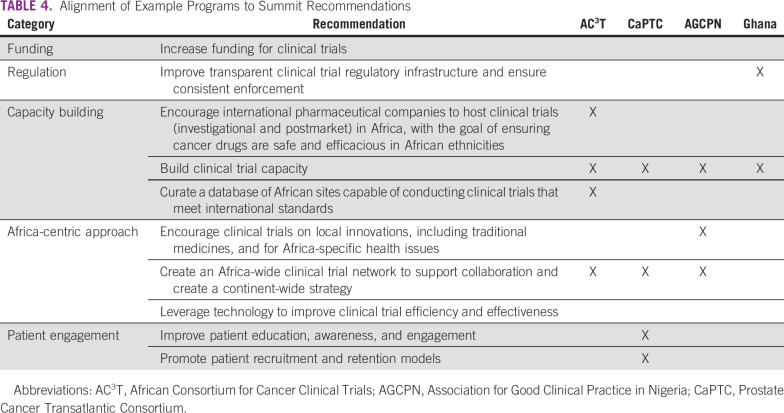
Alignment of Example Programs to Summit Recommendations

Although this article describes several programs that are improving Africa’s clinical trial environment, it is important to note that this is not an exhaustive review of such programs. Indeed, many other programs, such as the EDCTP, are improving clinical trial systems and infrastructure within Africa. Furthermore, none of the programs described above currently cover all African countries. As such, a concerted effort must be made to connect relevant initiatives, including those described in this article, to share best practices, minimize duplication, and ensure even coverage across Africa. To this end, BVGH has shared its AC^3^T Checklist with the administrators of AGCPN and CaPTC to ensure their sites are profiled and promoted online. Profiled sites not in compliance with GCP standards will be connected with CaPTC and encouraged to use its CCaRES program. AGCPN, BVGH, and CaPTC will jointly explore the cultivation of online training programs covering topics identified as frequently lacking via the AC^3^T Checklist. Last, to ensure these activities improve the number of rigorous cancer clinical trials performed across Africa, the involvement of the African Organization for Research and Training in Cancer in these programs will be explored.

It is also important to note that, although the two summits highlight factors that are likely to affect Africa’s future participation in clinical trials, they do not comprehensively capture all facets of the continent’s clinical trial landscape. Geographically, both summits were limited in representation and scope. The Operational Strategy for Clinical Trials in Nigeria Summit focused on Nigeria’s clinical trial challenges and opportunities. The 1st All Africa Clinical Trial Summit involved participants from a limited number of African countries. Both summits plan to expand their involvement of additional African countries in the future. In addition, neither summit systematically reviewed and debated all possible factors that affect Africa’s clinical trial participation, yet neither summit was organized with this goal in mind. In the absence of a comprehensive assessment of Africa’s clinical trial challenges and limitations, the recommendations of these two summits’ participants will help to highlight areas of need and opportunities to make a significant impact on the ability of the African continent to attract and conduct clinical trials, including those focused on new cancer interventions.

Cancer mortality rates in high-income countries, such as the United States and Canada, have declined significantly over the past three decades.^31,32^ These falling mortality patterns are due, in large part, to the development of new innovative cancer treatments. Unfortunately, with experts predicting Africa’s cancer mortality rate to double by 2040, Africans are not reaping the benefits of these innovations to the same degree as other populations.^33^ Ensuring proportionate enrollment of individuals of African descent in clinical trials will undoubtedly contribute to the reversal of Africa’s disturbing cancer trends.

Novel strategies, incentives, and programs are essential to increasing the number of clinical trials conducted in individuals of African descent—in particular, those living in Africa. The two summits and four programs described in this article have highlighted several of the challenges Africa faces in increasing its clinical trial participation and, more importantly, concrete Africa-driven solutions to those challenges. These solutions include building capacity across all levels of the cancer care system. Governments must play an active role by establishing regulatory guidelines and appointing the appropriate entity to enforce those guidelines. Connectivity is key; the augmentation of Africa’s clinical trial output will require the coordinated engagement of all relevant stakeholders, including African government agencies and cancer clinicians, pharmaceutical companies, international cancer experts, and patients with cancer. Improved visibility of Africa’s cancer burden and available clinical trial infrastructure will catalyze the pharmaceutical industry’s interest and demystify entry into the African clinical trial field. Last, and most importantly, collaboration, evaluation, and the sharing of lessons learned will drive clinical trial growth across the continent and, eventually, result in improved outcomes for patients with cancer in Africa and beyond.

## Figures and Tables

**TABLE A1:**
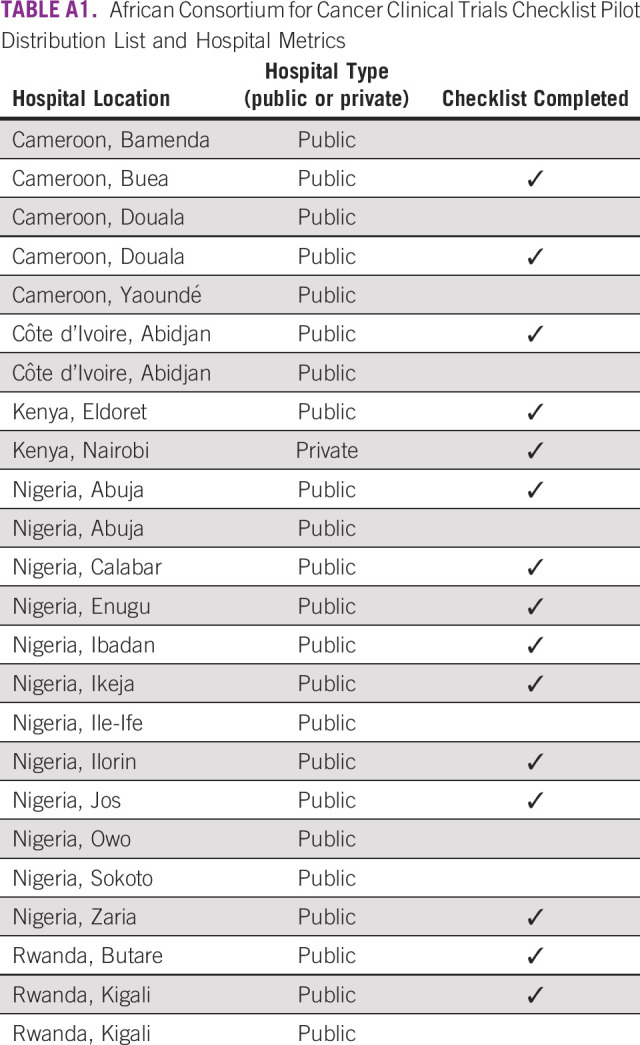
African Consortium for Cancer Clinical Trials Checklist Pilot Distribution List and Hospital Metrics
